# Comparison of 30-Day Readmission Between Early and Late Catheter Directed Thrombolysis for Acute Pulmonary Embolism in the United States

**DOI:** 10.3390/jcdd12040118

**Published:** 2025-03-27

**Authors:** Kwabena Sefah Nketiah Sarpong, Sun-Joo Jang, Mary Quien, Karthik Muralidharan, Abhinav Aggarwal, Ilhwan Yeo, Kavitha Gopalratnam

**Affiliations:** 1 Division of Pulmonary, Critical Care and Sleep Medicine, Department of Medicine, Icahn School of Medicine at Mount Sinai, New York, NY 10029, USA; knketiahsarpong@gmail.com; 2 Section of Cardiovascular Medicine, Department of Internal Medicine, Yale University School of Medicine, 20 York St, New Haven, CT 06510, USA; 3 Division of Cardiology, Department of Internal Medicine, Yale New Haven Health, Bridgeport Hospital, Bridgeport, CT 06610, USA; mmquien@gmail.com; 4 Department of Internal Medicine, Yale New Haven Health, Bridgeport Hospital, Bridgeport, CT 06610, USA; karthikmdharan@gmail.com (K.M.); draggarwalabhinav@gmail.com (A.A.); 5 Division of Pulmonary and Critical Care Medicine, Department of Medicine, Mayo Clinic, Rochester, MN 55905, USA; iy2159@caa.columbia.edu; 6 Division of Pulmonary/Critical Care Medicine, Department of Medicine, Yale New Haven Health, Bridgeport Hospital, Bridgeport, CT 06610, USA; kavithagr@gmail.com

**Keywords:** pulmonary embolism, catheter directed thrombolysis, mortality; readmission

## Abstract

Background: Pulmonary embolism (PE) is a major cause of morbidity and mortality accounting for 100,000 deaths per year in the United States and 5–10% of in-hospital deaths. There is sparse comparative data on readmission patterns in patients who undergo early versus late catheter directed thrombolysis (CDT) for acute PE. Research Question: Is the procedure day for acute PE associated with a difference in 30-day readmission rates? Study Design and Method: This study was performed by using the Nationwide Readmissions Database between 2016 and 2019. Patients with acute PE who underwent CDT were identified using codes from the International Classification of Diseases, 10th Edition (ICD 10). Results: The 30-day readmission rates were higher for patients who underwent CDT on Day 2 or afterwards compared to Day 1 and Day 0 (7.1% vs. 5.7% vs. 5.2%). Patients who had CDT on Day 2 or later had a higher rate of 30-day readmission mortality compared to those who had CDT on Day 1 or 0 (0.6% vs. 0.3% vs. 0.2%. The 30-day readmission rates for heart failure were higher among patients who had CDT on Day 2 compared to those who had CDT on Day 0 of admission (2% vs. 1.0% vs. 0.9%). Interpretation: Delayed CDT for acute PE was associated with increased rates of 30-day readmission, readmission mortality, and readmission for heart failure. These findings emphasize the need for earlier CDT for the treatment of acute PE.

## 1. Introduction

Acute pulmonary embolism (PE) is the third most common cause of cardiovascular mortality in the United States [1]. It accounts for 100,000 deaths per year in the United States and 5–10% of in-hospital deaths [2]. Patients can present in a heterogenous fashion ranging from asymptomatic to hemodynamic instability and death. The risk classification for PE is dependent on the hemodynamic consequences of the embolus and has a strong relationship with outcomes [3]. While anticoagulation has remained the standard for the treatment of PE, the acute management of PE has evolved to systemic thrombolytic therapy, catheter directed thrombolysis (CDT), and catheter or surgical thrombectomy.

CDT was developed as an alternative to systemic thrombolysis and involves localized delivery of a lower dose thrombolytic agent. Meta-analysis has shown reduced 30-day and 1-year all-cause mortality compared to anticoagulation and reduced all-cause hospital mortality and incidence of intra-cranial hemorrhage compared to systemic thrombolysis in patients with sub-massive PE who underwent CDT [4,5]. Randomized controlled trials and single arm studies have shown reduced RV dilation, decreased pulmonary hypertension, and minimized intra-cranial hemorrhage in patients who underwent CDT for acute PE [6,7]. The ULTIMA Trial demonstrated an improvement in right ventricular function with CDT compared to anticoagulation alone without increasing bleeding complications in patients with intermediate risk pulmonary embolism [6].

Although CDT is increasingly being performed for acute submassive PE in the United States, no guidelines currently exist regarding the optimal timing of CDT. In a review of six prior CDT studies that included time to procedure, Rawal et al. found a trend towards benefit with early interventions (<24–48 h after presentation) compared with delayed intervention (>48 h after presentation), with improvements in pulmonary arterial pressures, right ventricular (RV) to left ventricular (LV) ratios, with low rates of bleeding and low post procedural and in hospital mortality [8]. A study by Lehr et al. demonstrated that intermediate-to-high risk patients with pulmonary embolism who underwent CDT within 24 h of admission were more likely to have shorter hospital stays and ICU LOSs as well as improvements in hemodynamic parameters compared to those who underwent CDT after 24 h [9].

There is however sparse comparative data on early readmission rates in patients who undergo early versus late CDT for acute PE. We aimed to explore the association of procedure day with 30-day readmission rates in patients who underwent CDT for acute PE using a large administrative database in the US.

## 2. Methods

This study was performed by using the Nationwide Readmissions Database (NRD) between January 2016 and November 2019. The NRD is a large, administrative database constructed using discharge data from HCUP State Inpatient Databases [10]. It has verified patient linkage numbers used to track the patients across hospitals within a state during a given year. The NRD is designed to support national readmission analyses and is a publicly available and nationally representative healthcare database. Each patient record in the NRD contains information on the patient’s diagnoses and procedures performed during the hospitalization, based on the International Classification of Diseases, 10th Revision-Clinical Modification (ICD-10-CM) codes or Procedure Coding System (ICD-10-PCS). We identified our study population, comorbidities, causes of readmissions, and in-hospital outcomes using a combination of ICD-10-CM and ICD-10-PCS. Institutional Review Board approval and informed consent were not required for the current study because all the data collection was derived from a publicly open and deidentified administrative database.

From January 2016 to November 2019, Patients with acute PE who underwent CDT were identified using ICD-10-CM codes for PE (I26.02, I26.09, I26.92, I26.93, I26.94, and I26.99) and ICD-10-PCS codes for CDT (3E06017, 3E06317,6A75, 6A750, 6A750Z, 6A751, 6A751Z, 6A750Z6, 6A751Z6, 6A750Z7, 6A751Z7, 6A750ZZ, 6A751ZZ, 02CQ3ZZ, 02CP3ZZ, 02CP4ZZ, 02CQ0ZZ, 02CQ3ZZ, 02CQ4ZZ, 02CR0ZZ, 02CR3ZZ, and 02CR4ZZ). Patient- and hospital-level variables were included as baseline characteristics. Since NRD prohibits linking patients across years, patients whose index hospitalization was in December were excluded in order to allow for completeness of data on 30 days of follow-up after discharge, similar to other prior studies examining the NRD [11,12,13]. Patients who were <18 years old and patients who underwent thrombolysis for STEMI and ischemic stroke were also excluded. Patients were divided into three groups according to the procedure day (day 0, day 1, and day ≥2) and their baseline characteristics and outcomes were compared.

The primary outcome of interest was 30-day readmission rates after CDT for acute PE during index hospitalization. For 30-day readmissions, only the first readmission within 30 days of the discharge was included, and transfer to another hospital was not counted as a readmission. The secondary outcomes were 30-day rates of readmission-related mortality and readmission for heart failure.

All statistical analyses were performed using the SAS software, version 9.4 (SAS Institute, Cary, NC, USA), and the R statistical software, version 4.2.3 (www.R-project.org (accessed on 24 March 2023)), with its package “survey”. The discharge weight and stratum provided by the NRD were used for all analyses and thus all the reported numbers are weighted national estimates. Domain analysis was used for accurate variance calculations for subgroup analyses. All analyses accounted for the NRD sampling design by including hospital-year fixed effects based on hospital identification number. We compared baseline patient- and hospital-level characteristics for patients who had CDT for acute PE, stratified by time of intervention. Categorical variables are presented as frequencies and were analyzed using the Rao–Scott chi-square test. Continuous variables are shown as mean with standard error (SE) or median with interquartile range (IQR) and were tested using either the Mann–Whitney–Wilcoxon test or a survey-specific linear regression test. To evaluate the predictive value of the time of intervention and other covariates for primary and secondary outcomes, survey-specific univariate and multivariable generalized linear models were applied. Variables with *p* < 0.1 were included as initial covariates. Final parsimonious models were created via the manual removal of each covariate, based on Akaike information criterion, while ensuring each removal did not result in a >10% change in the measure of association for the primary predictor variable. Adjusted risks are presented in adjusted odds ratio (aOR), together with a 95% confidence interval (CI) and *p* value. All tests were two-sided with a *p* value of <0.05 considered statistically significant.

## 3. Results

**Figure 1:**
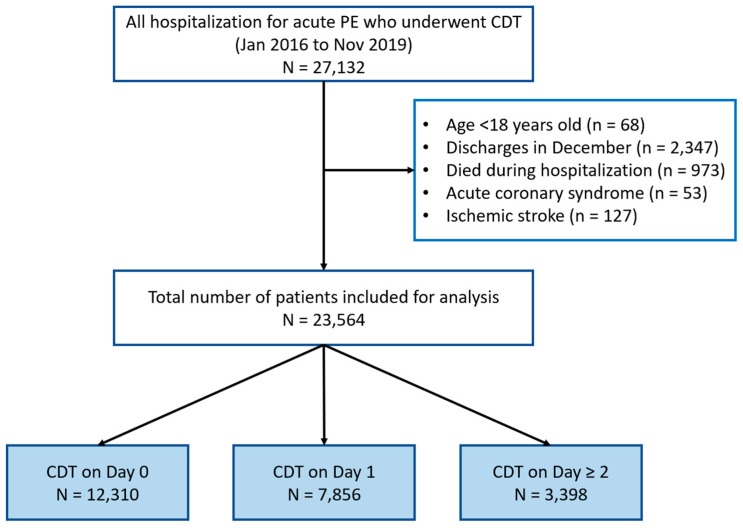
Flow diagram of study population.

Among a total of 23,564 patients who underwent CDT for acute pulmonary embolism, 12,528 (53.2%) were men and 11,034 (46.8%) were women (*p* = 0.014) (Table 1). The mean age of the patients was 60.3 years (SE 0.2). The median length of hospital stay was 4 days (IQR 3 to 6 days). Patients were divided into three groups according to their procedure day: 12,310 patients had theirs on day 0 (52.2%), 7856 patients had theirs on day 1 (33.3%), and 3398 patients had theirs on day ≥2 (14.4%) (Figure 1). The group of patients who had their procedure on day ≥ 2 were older and had more women.

**Figure 2:**
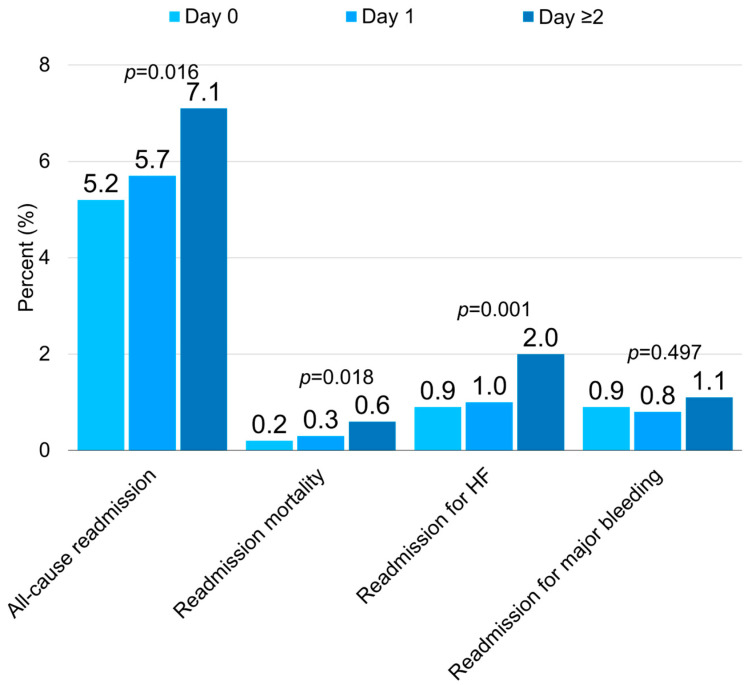
Thirty-day outcomes in patient with acute PE who underwent catheter-directed thrombolysis stratified by procedure day.

**Figure 3:**
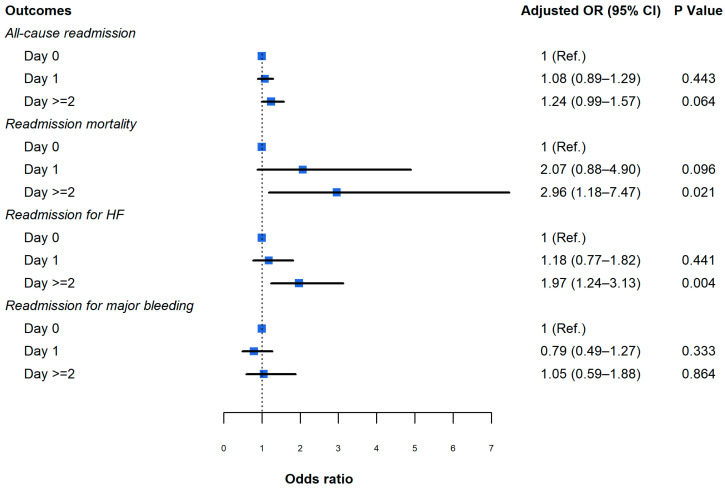
Adjusted association between procedure day and 30-day outcomes in patients with acute PE who underwent catheter-directed thrombolysis.

The 30-day readmission rates were higher for patients who underwent CDT on Day 2 or afterwards compared to Day 1 and Day 0 (7.1% vs. 5.7% vs. 5.2%, for day 2, day 1 and day 0, respectively; *p* = 0.016) (Figure 2, Supplementary Table S1). After multivariable risk adjustment, the 30-day risk for all-cause readmission was substantially higher among patients with CDT day ≥2 compared to those with CDT day 0 (aOR 1.24; 95% CI 0.99–1.57; *p* = 0.064) (Figure 3, Supplementary Table S2).

Patients who had CDT on day ≥2 had a higher rate of 30-day readmission mortality compared to those who had CDT on day 1 or 0 (0.6% vs. 0.3% vs. 0.2%, for day ≥2, day 1 and day 0, respectively; *p* = 0.018). The 30-day readmission rate for heart failure was higher among patients who had CDT on day ≥2 vs. those who had CDT on day 0 of admission (2% vs. 1.0% vs. 0.9%, for day ≥2, day 1 and day 0, respectively). The 30-day adjusted risk for readmission-related mortality was higher in patients with CDT on day ≥2 compared to those who had CDT on day 0 (aOR 2.96; 95% CI 1.18 to 7.47; *p* = 0.021). The 30-day adjusted risk for readmission for heart failure was also higher in patients with CDT day ≥2 compared to those who had CDT on day 0 (aOR 1.97; 95% CI 1.24 to 3.13; *p* = 0.004). There was no significant difference in the incidence of major bleeding during the 30 days after discharge (1.1% vs. 0.8% vs. 0.9%, for day ≥2, day 1 and day 0 respectively; *p* = 0.497).

## 4. Discussion

Using the large administrative data in the US, we identified several key findings for early outcomes in patients with acute PE who underwent CDT. Delayed CDT (day 2 or later) for acute PE was associated with increased rates of 30-day readmission compared to CDT procedures that took place on day 0 or day 1. Delayed CDT was also associated with an increased 30-day risk of readmission mortality and readmission for heart failure. These findings emphasize clinical and economic benefits for the earlier CDT for the treatment of acute PE.

A delay in therapeutic procedures can directly affect effective healthcare delivery by prolonging the length of stay, increasing hospital costs, and increasing adverse effects in patients. Chao et al. reported that for each additional day of symptoms before initiating thrombolysis, there was a decrease of 0.8% in lung tissue reperfusion observed on lung scans [14]. Various factors may contribute to delayed diagnosis and treatment of patients with acute PE. The early recognition of pulmonary embolism and effective time sensitive planning of interventions play a major role in preventing the delay of catheter directed thrombolysis. A study conducted in China examined the correlation between early thrombolysis and clinical parameters, revealing that the presence of bedside clinical signs such as a cough, crackles, a murmur in the tricuspid area, and an increased P2 were associated with earlier recognitions of pulmonary embolism [15]. Also, there are multiple stages involved in CDT planning which include appropriate patient selection, pre-procedure evaluation, the CDT procedure, and post-procedure care. Procedure timings can be affected by complex interactions between the various services involved in the patient’s care, such as the primary team, nurses, interventional radiologists, cardiologists, or critical care specialists.

The impact of the procedure day on the outcomes of CDT has not been widely studied. A recent study showed patients who underwent CDT within 24 h of admission were more likely to have shorter hospital and intensive care unit (ICU) lengths of stay [9]. The 30-day readmission rate for patients who underwent CDT in our study was 5.6% similar to other studies [16,17]. Our study demonstrates that delayed CDT is associated with significant risk for readmission after index hospitalization for acute PE. In addition to delayed CDT, several other factors have been deemed to be associated with readmissions after index admission for PE. These include African Americans, patients without private health insurance, and those discharged home with supplemental care or who left the hospital against medical advice as well as more severely ill patients (i.e., those with comorbid conditions and signs of cardiorespiratory instability) [18]. It is imperative therefore that follow up in both the inpatient and outpatient settings is carefully planned to avert readmissions with particular attention to patients with the above-named risk factors.

Our study demonstrated increased readmission mortality and readmissions for heart failure with delayed CDT compared to early CDT. Hemodynamic and echocardiographic parameters, including pulmonary arterial systolic pressure (PASP) and left ventricle stroke volume (LVSV), have been shown to predict mortality in acute PE. Kamran et al. showed that simultaneously assessing PASP and LVSV as a ratio PASP/LVSV accurately predicts adverse clinical events, including death, cardiogenic shock, and the need for advanced interventional procedures [19]. Lehr et al. demonstrated that patients who underwent CDT within 24 h of admission had modest improvements in pulmonary arterial systolic pressure (PASP), which is an important initial pathophysiologic risk factor that drives right ventricular dysfunction in PE and has been shown to predict mortality [9,19]. Within the first 72 h of acute pulmonary embolism, right ventricular (RV) failure plays a significant role in mortality, with approximately 45% of patients experiencing some degree of RV dysfunction [20]. Patients with right heart dilatation and dysfunction tend to have larger clot volumes, leading to variable degrees of pulmonary vascular obstruction [21]. However, the relationship between clot volume on imaging and adverse outcomes related to RV failure has been inconclusive [22]. Another recent study investigated the timing of ultrasound-guided CDT in acute pulmonary embolism and demonstrated that early CDT (administered within 24 h of diagnosis) resulted in a significant improvement in cardiac index, pulmonary vascular resistance, and mean right ventricular stroke work index compared to delayed CDT (administered after 24 h) [23].

The ULTIMA Trial also demonstrated that early interventions with CDT within 4 h of baseline echocardiography led to improvements in the RV/LV ratio, which is an important predictor of mortality [6]. These pathophysiologic mechanisms and the improvement in hemodynamic parameters with early CDT may underlie the differences in outcomes, particularly readmission mortality and readmissions for heart failure among the two groups.

This study has several limitations. First, this study is limited by its retrospective design. Second, since data was extracted from an administrative database, we are unable to derive further details from patient cases. The study, thus, does not account for other patient specific or clinical variables that may have contributed to the differences we found between our cohorts. For example, hemodynamic parameters such as the PASP, which are known to predict morbidity and mortality, could not be derived from the NRD. Lastly, the NRD is only able to provide the day of procedure and not the specific time the procedure took place in terms of hours. This likely led to overlap between the groups, which may have affected the outcomes.

To summarize, our study found that patients with acute PE who were treated with CDT within 24 h were associated with decreased rates of 30-day readmission, readmission mortalities, and readmissions for heart failure when compared to those who received delayed CDT. While more robust studies are needed to confirm these findings and to understand their benefits in different patient subgroups, our study emphasizes the benefits of early CDT in the management of acute PE. Further studies implementing a “door-to-catheterization time” for CDT may lead to more detailed differences in clinical and economic outcomes [8].

## Figures and Tables

**Table 1:** Patient and hospital characteristics for patients hospitalized with acute pulmonary embolism who underwent catheter-directed thrombolysis.

Characteristics	Overall	Day 0	Day 1	Day ≥2	*p* Value
Number of patients, n (%) *	23,564	12,310 (52.2)	7856 (33.3)	3398 (14.4)	
**Patient characteristics**					
**Age, mean (SE), y**	60.3 (0.2)	59.9 (0.2)	60.6 (0.3)	61.5 (0.4)	<0.001 †
**Gender**					0.014 ‡
**Male**	12,528 (53.2)	6678 (54.3)	4148 (52.8)	1702 (50.1)	
**Female**	11,034 (46.8)	5631 (45.7)	3708 (47.2)	1695 (49.9)	
**Smoking**	6080 (25.8)	3142 (25.5)	2073 (26.4)	865 (25.5)	0.636
**Hypertension**	12,632 (53.6)	6467 (52.5)	4310 (54.9)	1855 (54.6)	0.069
**Diabetes mellitus**	6391 (27.1)	3227 (26.2)	2132 (27.1)	1031 (30.4)	0.009
**Dyslipidemia**	7551 (32.0)	3765 (30.6)	2636 (33.6)	1150 (33.9)	0.004
**Family history of coronary artery disease**	1989 (8.4)	956 (7.8)	762 (9.7)	271 (8.0)	0.006
**Congestive heart failure**	1095 (4.6)	524 (4.3)	344 (4.4)	227 (6.7)	<0.001
**Coronary artery disease**	2750 (11.7)	1365 (11.1)	909 (11.6)	476 (14.0)	0.010
**Peripheral vascular disease**	1114 (4.7)	546 (4.4)	361 (4.6)	207 (6.1)	0.038
**Previous myocardial infarction**	805 (3.4)	413 (3.4)	230 (2.9)	162 (4.8)	0.013
**Previous stroke**	907 (3.8)	444 (3.6)	324 (4.1)	139 (4.1)	0.489
**Previous CABG**	500 (2.1)	283 (2.3)	155 (2.0)	62 (1.8)	0.350
**Previous PCI**	711 (3.0)	331 (2.7)	280 (3.6)	100 (2.9)	0.070
**Chronic pulmonary disease**	4535 (19.2)	2182 (17.7)	1536 (19.5)	817 (24.1)	<0.001
**Pulmonary hypertension**	6218 (26.4)	2976 (24.2)	2084 (26.5)	1158 (34.1)	<0.001
**Pulmonary circulatory disorder**	6497 (27.6)	3109 (25.3)	2175 (27.7)	1213 (35.7)	<0.001
**Chronic kidney disease**	2295 (9.7)	1019 (8.3)	807 (10.3)	469 (13.8)	<0.001
**Liver disease**	947 (4.0)	440 (3.6)	332 (4.2)	175 (5.2)	0.012
**Anemia**	3950 (16.8)	1973 (16.0)	1279 (16.3)	698 (20.5)	<0.001
**Atrial fibrillation**	2231 (9.5)	1160 (9.4)	713 (9.1)	358 (10.5)	0.271
**Valvular heart disease**	1908 (8.1)	936 (7.6)	583 (7.4)	389 (11.5)	<0.001
**Coagulopathy**	3251 (13.8)	1671 (13.6)	1072 (13.6)	508 (15.0)	0.364
**Autoimmune disease**	822 (3.5)	401 (3.3)	294 (3.7)	127 (3.7)	0.431
**AIDS**	88 (0.4)	54 (0.4)	18 (0.2)	16 (0.5)	0.199
**Alcohol use disorder**	864 (3.7)	429 (3.5)	297 (3.8)	138 (4.1)	0.527
**Drug abuse**	466 (2.0)	219 (1.8)	177 (2.3)	70 (2.1)	0.273
**Obesity**	9761 (41.4)	5005 (40.7)	3200 (40.7)	1556 (45.8)	0.002
**Hypothyroidism**	2852 (12.1)	1370 (11.1)	1033 (13.1)	449 (13.2)	0.004
**Movement disorder**	534 (2.3)	279 (2.3)	184 (2.3)	71 (2.1)	0.862
**Seizure disorder**	603 (2.6)	302 (2.5)	2011 (2.7)	90 (2.6)	0.755
**Dementia**	578 (2.5)	269 (2.2)	222 (2.8)	87 (2.6)	0.121
**Depression**	2800 (11.9)	1339 (10.9)	9,24 (11.8)	537 (15.8)	<0.001
**Cancer**	1699 (7.2)	852 (6.9)	591 (7.5)	256 (7.5)	0.495
**Median household income**					0.477
**First quartile**	6932 (29.4)	3609 (29.3)	2248 (28.6)	1075 (31.7)	
**Second quartile**	6749 (28.6)	3500 (28.4)	2280 (29.0)	969 (28.5)	
**Third quartile**	5,91 (25.3)	3115 (25.3)	2023 (25.7)	823 (24.2)	
**Fourth quartile**	3920 (16.6)	2085 (16.9)	1306 (16.6)	529 (15.6)	
**Primary payer**					<0.001
**Medicare**	10,723 (45.5)	5399 (43.9)	3646 (46.4)	1678 (49.4)	
**Medicaid**	1903 (8.1)	986 (8.0)	579 (7.4)	338 (9.9)	
**Private including HMO**	8876 (37.7)	4804 (39.0)	2929 (37.3)	1143 (33.6)	
**Self-pay/no charge/other**	2062 (8.8)	1121 (9.1)	703 (8.9)	238 (7.0)	
**Weekend admission**	5493 (23.3)	2597 (21.1)	1882 (24.0)	1014 (29.8)	<0.001
**Cardiogenic shock**	603 (2.6)	369 (3.0)	168 (2.1)	66 (1.9)	0.007
**Cardiac arrest**	180 (0.8)	106 (0.9)	60 (0.8)	14 (0.4)	0.214
**Length of hospital stay, d (IQR)**	4 (3–6)	4 (4–5)	4 (4–5)	6 (6–7)	<0.001
**Hospital characteristics**					
**Hospital teaching status**					0.015
**Teaching**	17,755 (75.3)	9452 (76.8)	5797 (73.8)	2506 (73.8)	
**Nonteaching**	5809 (24.7)	2858 (23.2)	2060 (26.2)	891 (26.2)	
**Hospital location**					0.002
**Rural**	11,785 (50.0)	6408 (52.1)	3807 (48.5)	1570 (46.2)	
**Urban**	11,777 (50.0)	5901 (47.9)	4049 (51.5)	1827 (53.8)	
**Hospital bed size**					0.558
**Small**	2499 (10.6)	1273 (10.3)	899 (11.4)	327 (9.6)	
**Medium**	6236 (26.5)	3277 (26.6)	2062 (26.2)	897 (26.4)	
**Large**	14,828 (62.9)	7760 (63.0)	4895 (62.3)	2173 (64.0)	
**Disposition**					<0.001
**Home**	17,527 (74.4)	9411 (76.5)	5857 (74.6)	2259 (66.5)	
**Facility ‖**	5979 (25.4)	2867 (23.3)	1982 (25.2)	1130 (33.3)	
**AMA/unknown**	56 (0.2)	31 (0.3)	17 (0.2)	8 (0.2)	

Abbreviations: SE, standard error; CABG, coronary artery bypass graft; PCI, percutaneous coronary intervention; AIDS, acquired immunodeficiency syndrome; HMO, health maintenance organization; AMA, against medical advice. * Values are presented as number (percentage) of patients unless otherwise indicated. † Survey-specific linear regression was performed. ‡ Rao–Scott χ^2^ test was used for all statistical tests unless stated otherwise. ‖ Facility includes skilled nursing facility, intermediate care facility, and inpatient rehabilitation facility.

## Data Availability

The data presented in this study are available on request from the corresponding author. Original United States Nationwide Readmissions Database used for this study is available at https://www.hcup-us.ahrq.gov/db/nation/nrd/nrddbdocumentation.jsp (accessed on 1 May 2024).

## Supplementary Materials

The following supporting information can be downloaded at: https://www.mdpi.com/article/10.3390/jcdd12040118/s1, Table S1: Thirty-day outcomes in patient with acute pulmonary embolism who underwent catheter-directed thrombolysis stratified by procedure day. Table S2: Adjusted association between procedure day and 30-day outcomes in patients with acute pulmonary embolism who underwent catheter-directed thrombolysis.
