# Supplementary material for: Comparison of 30-Day Readmission Between Early and Late Catheter Directed Thrombolysis for Acute Pulmonary Embolism in the United States
Source: J Cardiovasc Dev Dis. 2025 Mar 27;12(4):118. doi: 10.3390/jcdd12040118 (PMC12027995; doi:10.3390/jcdd12040118)
Supplement: Supplementary file 1 [file jcdd-12-00118-s001.zip › jcdd-3479042-supplementary.pdf]

Supplementary data

Table S1. Thirty-day outcomes in patient with acute pulmonary embolism who underwent catheter-directed thrombolysis stratified by procedure day

Table S2. Adjusted association between procedure day and 30-day outcomes in patients with acute pulmonary embolism who underwent catheter-directed thrombolysis

Table S1. catheter-directed thrombolysis stratified by procedure day

**Characteristics**	**Overall, n (%)**	**Day 0**	**Day 1**	**Day ≥2**	**P value**
**No. of patients***	23,564	12,310 (52.2)	7,856 (33.3)	3,398 (14.4)	
**Readmission**	1,328 (5.6)	641 (5.2)	446 (5.7)	241 (7.1)	0.016
**Readmission mortality**	66 (0.3)	20 (0.2)	27 (0.3)	19 (0.6)	0.018
**Readmission for HF**	256 (1.1)	108 (0.9)	81 (1.0)	67 (2.0)	0.001
**Readmission for major bleeding**	214 (0.9)	115 (0.9)	61 (0.8)	38 (1.1)	0.497

Abbreviations: HF, heart failure

*Analyses for 30-day follow up excluded population who died during the index hospitalization.

Table S2. Adjusted association between procedure day and 30-day outcomes in patients with acute pulmonary embolism who underwent catheter-directed thrombolysis

**Characteristics**	**Adjusted OR**	**95% CI (lower)**	**95% CI (higher)**	**P Value**
**Readmission**				
**Day 0**	1 (Ref)			
**Day 1**	1.08	0.89	1.29	0.443
**Day ≥2**	1.24	0.99	1.57	0.064
**Readmission mortality**				
**Day 0**	1 (Ref)			
**Day 1**	2.07	0.88	4.90	0.096
**Day ≥2**	2.96	1.18	7.47	0.021
**Readmission for HF**				
**Day 0**	1 (Ref)			
**Day 1**	1.18	0.77	1.82	0.441
**Day ≥2**	1.97	1.24	3.13	0.004
**Readmission for major bleeding**				
**Day 0**	1 (Ref)			
**Day 1**	0.79	0.49	1.27	0.333
**Day ≥2**	1.05	0.59	1.88	0.864
